# Genetic Polymorphisms in Systemic Lupus Erythematosus and Their Clinical Implications: A Narrative Review

**DOI:** 10.3390/ijms27093968

**Published:** 2026-04-29

**Authors:** Audrey Matilda Egan, Nor Adzimah Johdi, Elena Aisha Azizan, Rozita Mohd, Sakthiswary Rajalingham, Syahrul Sazliyana Shaharir, Mohamed Afiq Hidayat Zailani

**Affiliations:** 1 Department of Medicine, Faculty of Medicine, Universiti Kebangsaan Malaysia (UKM), Cheras 56000, Kuala Lumpur, Malaysia; p152939@siswa.ukm.edu.my (A.M.E.); sazliyana@ukm.edu.my (S.S.S.); 2 UKM Medical Molecular Biology Institute (UMBI), Universiti Kebangsaan Malaysia, Cheras 56000, Kuala Lumpur, Malaysia; 3 Department of Pathology, Faculty of Medicine, Universiti Kebangsaan Malaysia (UKM), Cheras 56000, Kuala Lumpur, Malaysia

**Keywords:** systemic lupus erythematosus, autoimmune disease, molecular genetics, genetic polymorphisms, single nucleotide polymorphism, targeted therapies, biomarkers

## Abstract

Systemic lupus erythematosus (SLE) is a polygenic autoimmune disorder where genetic diversity drives significant clinical heterogeneity. This review summarizes the current understanding of the roles of genetic polymorphisms in immunological dysregulation, organ-specific manifestations and therapeutic response heterogeneity in individuals with SLE. The literature was obtained from PubMed, EBSCOhost, Web of Science and Scopus. The narrative review comprised 60 publications published within the last 12 years. The research consistently identifies the major histocompatibility complex (MHC) region as the most significant genetic risk factor for the onset of autoimmunity. Genetic variants in *STAT4* and *IRF5* exacerbate disease progression by facilitating chronic inflammation. These genetic factors are associated with various clinical outcomes, including renal and neuropsychiatric symptoms. Polymorphisms in HLA class II, *TLR7* and *FBN2* are notably linked to serious consequences, including lupus nephritis (LN). Progress in targeted therapy signifies a transition to personalized medicine with medications such as anifrolumab, litifilimab, iberdomide and Janus kinase (JAK) or Cyclin-Dependent Kinase (CDK) inhibitors, demonstrating potential for targeting pathways associated with the interferon gene signature and *STAT4* polymorphisms. Notwithstanding the problems presented by the heterogeneity of SLE, the identification of risk variations is anticipated to enhance predictive and therapeutic biomarkers, hence facilitating more precise and individualized disease management.

## 1. Introduction

Systemic lupus erythematosus (SLE) is a chronic autoimmune disease characterized by the production of autoantibodies that attack the body’s own tissues and organs [1]. SLE primarily affects young women of reproductive age and carries a significant burden due to organ damage and disability caused by the disease [2,3,4,5]. The condition involves abnormal immune responses, especially those related to interferon (IFN) signaling [6], and an elevated Type I Interferon (IFN) signature is a hallmark of SLE that correlates with disease activity and organ involvement [7]. Advances in genetic research have shown that various pathways, including intra- and intercellular signaling pathways, the Toll-like receptor (TLR) signaling pathway, the JAK/STAT pathway, and the Nuclear Factor Kappa B (NF-κB) pathway, also contribute to the development of SLE. SLE can cause persistent inflammation, damaging multiple organs, such as the kidneys and skin, and presents with a wide range of symptoms, including thrombocytopenia, rash, vasculitis, arthritis, Lupus nephritis (LN), and neuropsychopathy [8,9,10].

Approximately 3.4 million people globally are affected by the disease, with over 400,000 new cases diagnosed each year. The highest incidence rates are observed in China, the United States, Barbados and Poland. The disease shows a significant 9:1 female predominance, especially during reproductive years, suggesting a potential hormonal influence in its development. This is further supported by findings that hormonal replacement therapy increases flare frequency in female and pregnant patients. Male patients tend to experience more severe symptoms, faster disease progression, greater renal involvement and failure, and a less favorable long-term prognosis, characterized by higher rates of hypertension, thrombosis, and hematological symptoms [1,2,3]. The disease burden varies greatly depending on socioeconomic status, with lower socioeconomic status (SES) regions in the USA showing the highest prevalence rates [4]. SLE has also substantially impacted overall productivity in both occupational and non-occupational activities based on a Malaysian multi-ethnic cohort study, and both impairments are significantly correlated with diminished quality of life [5].

Additionally, disparities in disease prevalence are evident across different ethnic groups, with non-European populations experiencing incidence rates that are 2- to 4-fold higher, earlier disease onset, and a 2- to 8-fold increased risk of end-stage renal disease (ESRD) compared to European populations [6]. Furthermore, Asian patients are also found to have higher rates of severe cardiovascular and renal complications. The estimated prevalence of SLE among Asians ranges from 30 to 50 per 100,000 individuals, with reports indicating that SLE prevalence is particularly higher among the Chinese population in Asia. In Malaysia, among the three major ethnic groups, Malays (55.1%), Chinese (24.3%), and Indians (7.4%), the prevalence of SLE is 43 per 100,000 within a total population of 22 million. The Chinese population exhibits the highest prevalence with 57 cases per 100,000, followed by Malays with 33 cases per 100,000, and Indians with 14 cases per 100,000 [7].

To date, over 300 SLE-associated gene loci have been identified through genome-wide association studies (GWASs), and these genes are involved in various pathways, including the signal transduction pathway in lymphocytes and immune complex processing, such as Type I (IFN) (*IRF7*, *BLK*), TLR (*PTPN22*), and NF-kB (*TNIP1*). Additionally, many other genes, including *STAT4*, *IRF5*, *KIR*, *IL1R*, *BLK*, *OX40L*, *FCGR2A*, *BANK1*, *SPP1*, *IRAK1*, *TNFAIP3*, *C2*, *C4*, *CIq*, *PXK*, *HLA-DR*, *PTPN22*, *AFF1,* and *MECP2,* are all known to be associated with the etiology of SLE [8,9].

Ongoing research indicates that our understanding of the intricate genetic architecture behind SLE manifestations remains limited. Genetic factors, including polymorphisms in various immune-related genes, significantly influence the severity and diverse clinical features of SLE. Recent studies suggest that specific genetic variants may affect the manifestation of SLE characteristics beyond renal involvement. GWASs have identified over 100 risk-associated single-nucleotide polymorphisms (SNPs) linked to SLE with strong statistical significance, *p* ≤ 5 × 10^−8^. Most of these SNPs individually provide only a slight increase in SLE risk, making them of limited clinical use for disease diagnosis. Numerous susceptibility loci have been identified across different SLE populations through genetic research, dating back to 1970. However, the genetic variation identified to date accounts for less than half of SLE’s heritability, with modest overall effect sizes (Odds ratio, OR ~1.5 to 1.2) [10]. A few additional significant signals, such as *STAT4* (OR = 1.5–1.7) or *IRF5* (OR ≈ 1.8–2.0), exist, although most SNPs have effects near 1.1–1.2. Additionally, GWAS-identified variants explain only a small part of SLE’s total heritability. Therefore, an unexplained hereditary component remains, potentially uncovered through epigenetics, but is not yet well understood in SLE. This highlights the importance of further exploring the genetic factors behind disease variability and developing personalized treatment approaches. The clinical complexity of SLE can lead to diagnostic delays, especially when the disease initially presents with atypical symptoms [11,12,13,14]. Due to its wide range of symptoms, SLE can be difficult to distinguish from other conditions with similar features, such as rheumatoid arthritis (RA) and myositis. Early diagnosis and treatment are essential for effectively managing SLE symptoms and reducing organ damage [11,12,13,15].

Clinical manifestations and comorbidities associated with SLE treatment can increase disease burden and lead to a variety of treatment options and outcomes. Even with current standard treatments, such as glucocorticoids and immunosuppressants, a new analysis of the international Definition of Remission in SLE (DORIS) cohort showed that only 50% of 1652 patients achieved remission or low disease activity [16]. One manifestation that affects drug disposition is renal involvement. Conditions such as LN and the presence of Antiphospholipid Syndrome (APS), along with treatments such as glucocorticoid use, should be considered in relation to the increased risk of cardiovascular development. Future research may indicate that using genetic information in clinical practice could fundamentally change how SLE is treated [17].

The current therapy options for SLE are pragmatic, focusing on developing composite biomarkers for daily use, which requires the robust development of novel therapeutic techniques and medications that target specific responsive pathways [18]. With several new genetic associations, functional studies, and clinical trials emerging in recent years, the field of SLE genetics is advancing rapidly.

This narrative review was conducted following the standards of the Scale for the Assessment of Narrative Review Articles (SANRAs) criteria [19]. A comprehensive search of the English-language literature from electronic databases and search engines, including PubMed, EBSCOhost, Web of Science (WoS), and Scopus, published between January 2014 and November 2025, was performed using relevant keywords, such as “genetics of SLE”, “genetic polymorphisms of SLE”, “genetics in SLE clinical implications”, and “biomarkers and therapeutic strategies”. Specifically, these keywords were combined using Boolean operators (AND, OR) as follows: “SLE genetic polymorphism” OR “SNPs” AND “clinical implications” OR “SLE gene polymorphisms” AND “disease severity”. Additionally, the review includes articles that could enhance understanding of SLE pathogenesis, disease phenotypes, diagnosis and future directions for therapy. These databases were supplemented with manual searches of the references within the included studies.

## 2. Genetic Drivers of Immune Dysregulation in SLE

SLE pathogenesis arises from interactions between environmental and genetic factors that lead to epigenetic changes, including DNA methylation and histone modifications. These alterations influence pro-inflammatory gene expression and drive disease progression. Genetic factors play a significant role, with heritability estimates ranging from 43% to 66% across different populations. Specifically, the heritability of SLE was found to be 43.9%, while shared (familial) and non-shared environmental factors contribute to 25.8% and 30.3% of susceptibility, respectively [20].

Gene polymorphism refers to differences in alleles that cause variation in the nucleotide sequence of a gene among individuals. This concept is crucial for understanding human susceptibility to disease and tolerance, as well as clinical symptoms and therapeutic responses. The most common type of DNA variation in humans is SNPs, which are single-nucleotide alterations [21,22]. These SNPs have been linked to SLE susceptibility and may contribute to disease development. Most SLE-related SNPs are located in non-coding regions (introns) and act as markers of co-segregation mostly in genes involved in immune responses [23]. Only a small portion of risk variants cause changes in protein sequences, such as in Fc receptors [24]. Over the last 20 years, numerous large-scale genetic studies have shown that genetic predisposition influences SLE onset, identifying about 100 loci associated with susceptibility and explaining much of its heritability. Individual polymorphisms have little clinical utility for disease identification, screening, or formal diagnosis due to the complex, polygenic nature of SLE. As of now, isolated SNPs cannot serve as independent diagnostic or prognostic markers due to insufficient effect sizes. Combining artificial intelligence (AI), machine learning, and genetic data with other omics datasets, such as transcriptomics and epigenomics, provides an opportunity to better understand how SLE risk variants affect molecular traits across different disease-relevant cell types and how they influence immune variability among individuals [2,25].

Genetic variants associated with disease risk can trigger activation of the Type 1 (IFN) pathway and lead to the production of pathogenic autoantibodies, including antiphospholipid antibodies, anti-double-stranded DNA (anti-dsDNA) antibodies, and anti-Smith (anti-Sm) antibodies, among others. Additional genetic associations indicate that immune system regulation is influenced by cytosolic nucleic acid sensors and the inflammasome, with variations in *TNFAIP3,* which encodes A20, facilitating the activation of the NF-κB pathway [21,22]. Due to their substantial heterogeneity, immunoglobulin, T cell receptor, and MHC genes are postulated to play distinct roles in the etiopathogenesis of autoimmune diseases. The human leucocyte antigen (HLA) region is among the earliest recognized genetic associations with SLE, owing to its high degree of polymorphism. Furthermore, both the HLA region and *IRF5* demonstrate a notable sex–gene interaction in men with SLE, possessing a substantially higher frequency of risk alleles than women [11,23].

### 2.1. Innate and Adaptive Immune Dysregulation

In SLE, innate immune dysfunction arises from abnormal activation of the complement system, natural killer (NK) cells, neutrophils and plasmacytoid dendritic cells (pDCs). A key factor in autoimmunity is the failure to clear apoptotic debris and neutrophil extracellular traps (NETs). Dysregulation of these cells and pathways leads to loss of immune tolerance, persistent inflammation, and multi-organ involvement, all of which are characteristic of SLE. These uncleared nucleic acids are recognized by toll-like receptors (TLRs), particularly *TLR7* and *TLR9*, which trigger excessive Type 1 (IFN) production and stronger germinal center (GC) reactions. Although *TLR9* overexpression has been observed in the renal cells of individuals with LN, the clinical applicability of specific genetic variants, such as the *TLR7* rs3853839 polymorphism, is constrained by moderate effect sizes and inconsistent replication across diverse ancestral groups [21,24,26,27]. Ultimately, these intrinsic signals connect to adaptive immune dysregulation by activating autoreactive lymphocytes, resulting in the chronic inflammation and multi-organ damage typical of the disease.

**Figure 1:**
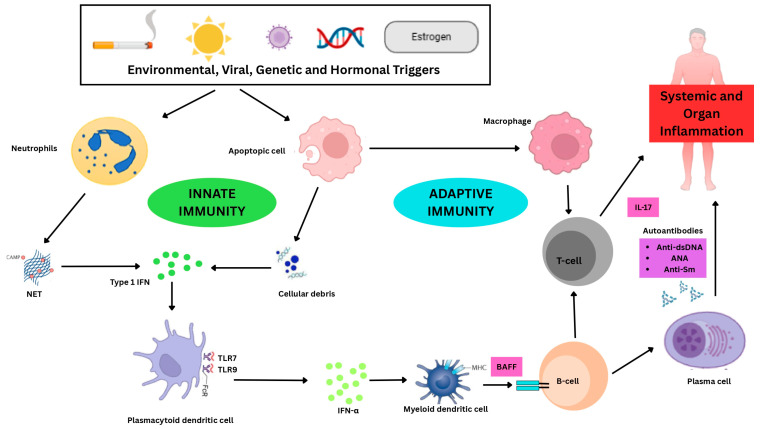
Innate and adaptive immune dysregulation in the pathogenesis of SLE. Predisposing factors for SLE, including genetic, epigenetic, viral, hormonal, and environmental influences, can stimulate the production of immunoreactants, leading to increased neutrophil apoptosis and NET formation, unbalanced macrophage M1 activation, and the initiation of TLR signaling (*TLR7*, *TLR-9*) in pDCs of the innate immune system. This enhances Type 1 (IFN) production or affects the growth and maturation of T and B cells in the adaptive immune system, leading to the overproduction of autoantibodies. Excess autoantibodies can form immune complexes that deposit in tissues, activate the complement system, and trigger inflammatory reactions.

Alongside innate triggers, adaptive immune responses in SLE are influenced by the breakdown of lymphocyte tolerance caused by particular genetic variants. The hyperactivity of B-cells and the resultant generation of pathogenic autoantibodies, such as anti-dsDNA and anti-Sm, are markedly affected by variations in *BLK* and *BANK1*, which impair B-cell receptor signaling and GC maturation. The dysregulation of T-lymphocytes is driven by the *STAT4* risk allele specifically rs7574865, which has been demonstrated to directly enhance mRNA and protein expression, thereby intensifying IL-12 and Type 1 (IFN) signaling. This genetic factor maintains a disrupted Th1/Th2 equilibrium and increased Th17 levels, promoting neutrophil recruitment and chronic tissue inflammation in the epidermis and kidneys [17,28]. The complex interactions between genetic risk variants and their respective innate and adaptive immune pathways, alongside their associated clinical manifestations, are synthesized in Table 1. Overall immunopathogenesis of SLE is summarized in Figure 1. 

### 2.2. Human Leukocyte Antigen (HLA) Genes of the Major Histocompatibility Complex (MHC) Region

The major histocompatibility complex (MHC) region on chromosome 6p21 is the most variable locus in the human genome among SLE loci. Strong correlations exist between SLE susceptibility and various autoantibodies across all populations in the MHC region. The MHC contains over 120 genes, including MHC class I genes (HLA-A, -B, -C, -E, -F, and -G) and class II genes (HLA-DP, -DM, -DO, -DQ, and -DR), as well as class III genes (complement components and others), many of which are essential for both innate and adaptive immune responses. To date, HLA genes have shown the strongest association with autoimmune diseases [29,30,31].

The HLA region contains about 200 genes, many of which have specific roles in the immune system. It exhibits considerable polymorphism, especially for HLA class II alleles, with varying frequencies reported across different ancestral groups. These alleles are associated with several clinical conditions, including LN [14,32,33]. HLA-DR2 (HLA-DRB115:01) and HLA-DR3 (HLA-DRB103:01), with an OR of 1.87, are strongly linked to SLE. These alleles show the strongest associations with SLE susceptibility across populations of European, Asian, North American, Central American and South American origins [32,34].

*HLA-DR3* has been linked to LN, specifically proliferative nephritis (Class III and IV), which is the most severe form of renal involvement. *HLA-DR3* is also associated with anti-dsDNA and anti-Ro/SSA autoantibodies, both of which are connected to more severe disease features and manifestations. In contrast, *HLA-DR2* is linked to anti-Sm antibodies, a common feature of SLE. Genotyping *HLA-DR* alleles, especially *HLA-DR2* (HLA-DRB115:01) and *HLA-DR3* (HLA-DRB103:01), could be a valuable tool for identifying high-risk patients with SLE, particularly those at risk of LN and severe organ damage [32].

In a recent study investigating the link between *HLA-DR2* and increased susceptibility to SLE in Taiwanese patients by Chang-Yi and colleagues, it was demonstrated that there is a significant difference in *HLA-DR2* levels between patients and controls (41.9% vs. 26.0%; OR = 2.05, 95% CI = 1.44–2.92, *p* < 0.001, adjusted *p* < 0.010). *HLA-DR2* carriers experienced an earlier disease onset and faced a higher risk of mouth ulcers, avascular necrosis (osteonecrosis) and LN [35]. Another study supporting the association between *HLA-DR2* and SLE was reported by Selvaraja et al., who found that carriers of HLA-DRB1*15 (OR = 1.56, 95% CI = 1.15–2.12) among Malay patients with SLE were more likely to present with mouth ulcers. Similarly, Bang et al. reported that HLA-DRB1*15:01 (OR = 1.88, 95% CI = 1.60–2.22) is associated with oral ulcers and renal involvement in the Korean population [11,36,37].

Additionally, a study by Indian researchers found that DRB1*0301 and DRB1*0701 showed a significant positive association among the HLA-DRB1 alleles analyzed in patients with SLE and controls. Conversely, DRB1*0401, DRB1*1401, DRB1*1404, and DRB1*1501 were significantly less common in patients with SLE. Similarly, DQB1*0202 and DQB1*0301 were significantly higher in SLE cases compared to controls, whereas DQB1*0501 was significantly lower in SLE. DQα1*0201 demonstrated a notable negative correlation with SLE. HLA-DRB1*0301 is frequently identified as a genetic risk factor in most Western and Indian studies. Another study conducted in Saudi Arabia revealed that HLA-DRB3 provides protection against SLE. Despite this, the study presented conflicting results, which is noteworthy, given Saudi Arabia’s high prevalence of consanguineous marriages [38].

### 2.3. Genotype-Phenotype Associations with SLE Clinical Manifestations

SLE-associated variations were correlated with the most common organ symptoms. A risk effect (OR) greater than 1, with a significant *p*-value less than 0.05, indicates that SNPs are associated with an increased risk of SLE, whereas a risk effect (OR) less than 1, with a significant *p*-value of less than 0.05, suggests that SNPs are associated with a reduced risk of SLE [34]. As a multigenic disease, numerous weighted genetic risk scores (GRSs) have been constructed to evaluate an individual’s cumulative genetic susceptibility to SLE, with higher GRSs associated with earlier SLE onset and elevated disease activity. Recent studies have shown significant correlations between a high GRS for the disease and the emergence of cardiovascular and renal manifestations, including a two-fold increase in the risk of LN and a five-fold increase in the risk of ESRD, in patients with a high GRS compared to those in the low quartile. In general, male patients with SLE have higher GRSs than female carriers, suggesting that genetic factors play a more dominant role in predisposing males to SLE susceptibility [36].

#### 2.3.1. Interferon Regulatory Factors (*IRFs*) Gene Polymorphisms

A family of transcription factors called interferon regulatory factors (*IRFs*) regulates various innate and adaptive immune responses, including immune cell differentiation, antiviral responses, and pro-inflammatory reactions to infections. *IRF5*, located on chromosome 7q32, encodes a transcription factor with a DNA-binding domain and a polymorphic region. *IRF5* regulates immune responses downstream of several pattern recognition receptors, including TLRs (TLR-3, 4, 7, 8 and 9), nucleotide-binding oligomerization domain 2 (NOD2), retinoic acid-inducible gene I (*RIG-I*) and dectin-1. Although *IRF5* has a broad expression pattern, it is consistently present in B cells, myeloid dendritic cells, pDCs, and monocytes/macrophages, where it stimulates the production of various pro-inflammatory cytokines and chemokines. *IRF5* influences both immunoglobulin class switching and GC development in B cells. Given its genetic association with SLE and its essential role in innate and adaptive immune responses contributing to SLE pathogenesis, *IRF5* is a potential therapeutic target for SLE [11,37,38].

According to Dahham and colleagues, the *IRF5* gene was significantly overexpressed in PBMCs from patients with SLE in a case–control study, resulting in increased T1FN expression. Hematological abnormalities are observed in the majority of patients with SLE, as shown by studies across diverse populations. The most common hematological manifestation is anemia, which is usually caused by a chronic disease. During disease flares and infections, patients with SLE have considerably higher erythrocyte sedimentation rate (ESR) values than healthy controls, which correlates with disease activity. The ESR is elevated due to reduced plasma protein concentrations and altered erythrocyte surface characteristics, which facilitate cell aggregation. These events may explain the negative association between hemoglobin and circulating *IRF5* protein, and a positive correlation between *IRF5* and ESR levels. These findings indicate that high *IRF5* expression is associated with ongoing inflammation and renal involvement, consistent with its role in amplifying T1FN signaling. Dahham’s findings support the notion that circulating *IRF5* serves as a biological marker of active and organ-involved SLE, particularly with respect to inflammatory and nephritic burden [29,39,40]. Additionally, genetic variants in *IRF5* are linked to the likelihood of developing SLE and are strongly associated with anti-Ro autoantibodies, which are among the most commonly identified autoantibodies against nuclear antigens and are also associated with SLE-associated Sjögren’s disease [30].

#### 2.3.2. Signal Transducer and Activator of Transcription 4 (*STAT4*)

Signal transducer and activator of transcription 4 (*STAT4*) is one of the most important SLE risk loci outside the HLA region, with the gene’s third intron harboring the most significant SNPs. *STAT4* influences T-helper (Th) type 1 and Th17 differentiation, monocyte activation, and IFN-ɣ production, and has been shown to regulate interleukin (IL)-12, IL-23, and Type 1 (IFN) cytokine signals in T-cells and monocytes. The *STAT4* SNPs associated with SLE are linked to an earlier disease onset, more severe disease phenotype, and increased risk of stroke and LN with severe renal insufficiency. In European populations, *STAT4* polymorphisms have been associated with severe LN. A GWAS study was conducted to investigate genetic association with LN in two Swedish cohorts, revealing genome-wide significant associations (*p* < 5 × 10^−8^) at four SNPs within the *STAT4* gene. Furthermore, an association with *STAT4* was identified in patients with SLE experiencing severe renal insufficiency (*p* = 7.6 × 10^−6^)[28].

A study by Lamana et al. in a Spanish SLE population found that *STAT4* mRNA and protein levels are significantly elevated and functionally linked to the minor T allele of rs7574865, a well-known genetic risk factor for autoimmune disorders, such as SLE and RA. In particular, the patients homozygous for the risk allele (TT) showed higher protein expression than those with the wild-type genotype in a cohort of patients with early arthritis, indicating that this variant directly upregulates *STAT4* transcription and translation. Interestingly, the patients with the risk allele had lower serum IL-6 levels, despite similar levels of inflammation (CRP). This suggests a distinct pathogenic mechanism in which increased STAT4 abundance likely amplifies downstream signaling, such as the IL-12 or T1FN pathways, rather than relying on IL-6-driven inflammation [41].

### 2.4. Ancestry-Specific Genetic Architecture and Phenotypic Heterogeneity

SLE can range from mild to potentially life-threatening, and its clinical manifestations can be highly heterogeneous among individuals, especially regarding organ involvement. LN is the most common and serious organ complication of SLE, affecting approximately 30–60% of adults and up to 70% of adolescents with the condition. It has led to higher long-term treatment costs, increased morbidity, and reduced life expectancy. Despite current treatments, only a small percentage of patients achieve remission from LN. Research by Chen et al. revealed that the onset of LN is not linked to any particular causal loci but is positively correlated with the genetic burden of SLE risk variants. This indicates that the heightened severity of SLE is attributable to a substantial burden of SLE causal alleles, rather than to a distinct set of risk genes. LN has been strongly associated with genetic variations in *ITGAM*, *TNFSF4*, *APOL1*, *PDGFRA,* and *SLC5A11,* among others [7,42,43].

In their study of Egyptian patients with SLE, Azab and colleagues found that the *TLR7* rs3853839 CG genotype differed significantly from the wild-type CC genotype (*p* = 0.04; OR = 0.53; 95% CI = 0.30 to 0.98). The *TLR7* rs3853839 CG and wild-type genotypes were associated with oral ulcers, a commonly reported clinical feature of SLE. Additionally, a significant correlation was observed between the *TLR7* rs3853839 G allele and thrombocytopenia (C vs. G, *p* < 0.0001; OR = 0.04, 95% CI = 0.01–0.16). This finding aligns with previous research by Raafat et al., who identified notable differences in the distribution of the *TLR7* rs3853839 genotypes between patients with SLE and controls. The polymorphic genotypes (CG and GG) were more prevalent in patients with SLE (60%) compared to healthy controls (34%). Supporting these findings, an extensive, multiethnic, multicenter study involving Korean, Chinese, and Japanese populations also identified the *TLR7* rs3853839 G/C polymorphism as a risk factor for SLE [43,44].

A GWAS SLE study by Tangtanatakul et al. in the Thai SLE population identified several SLE susceptibility genes, including variants in HLA class II, *STAT4*, *BLK*, *FAM167A-BLK*, *GTF21,* and a novel variant on the *FBN2* allele. Since Fibrillin-2 is a glycoprotein essential for bone, muscle, and extracellular matrix formation, mutations in *FBN2* cause dominant heritable connective tissue disorders. The most significant association was observed in the HLA class II region, consistent with previous findings in other ethnic groups and strongly linked to SLE risk (*p* < 5 × 10^−8^). The *BLK* and *STAT4* loci were recently reported as SLE susceptibility alleles in Thai patients, but the *GTF2I* locus was discovered for the first time in the study. Notably, polymorphisms at the *STAT4* and *GTF2I* loci were associated with LN across various SLE populations. Thai patients with SLE might have increased susceptibility to fibrosis-related inflammation. Additionally, *FBN2* overexpression has been linked to fibrosis formation in spontaneous LN mouse models. While the exact role of *FBN2* in SLE remains unclear, existing evidence suggests that this variant could enhance either fibrosis-associated inflammation or inflammatory processes during disease progression [45].

NPSLE refers to the neurological and psychiatric symptoms observed in 80% of individuals with SLE. These symptoms affect the CNS and peripheral nervous system (PNS), presenting in both localized and broad forms with varying degrees of severity. Acute confusional state (ACS), anxiety, mood problems, cognitive dysfunction (CD), headache, and psychosis are prevalent symptoms. *TREX1* mutations are associated with elevated T1FN expression and heightened seizure vulnerability in SLE. Multiple SNPs on the *TREX1* gene, such as rs781221615, rs3135944, and rs11797, have been associated with NPSLE. A study determined that Japanese patients with SLE exhibited a markedly higher cumulative count of risk alleles across eight well-established susceptibility loci for SLE, namely *HLA-DRB1*, *IRF5*, *STAT4*, *BLK*, *TNFAIP3*, *TNIP1*, *FCGR2B,* and *TNFSF13,* compared to healthy controls. The patients with more than 10 risk alleles exhibited a greater propensity for neurological involvement, suggesting that a higher number of risk alleles may contribute to more severe disease manifestations [11,21].

Approximately 70% of all individuals with SLE have varying degrees of cutaneous involvement. Certain genetic variants appear to correlate with dermatological manifestations (e.g., *FCGR2* rs1801274 linked to malar rash), although only a singular monogenic causative mutation in the *TREX1* gene has been discovered. A recent study revealed that patients with SLE with *TREX1* mutations had the greatest photosensitivity to UVA exposure, accompanied by a significant increase in UVB exposure and sensitivity to solar simulated irradiation (SSR) after 48 and 72 h [11]. Table 2 below summarizes gene polymorphisms associated with clinical phenotypes across different ethnicities.

### 2.5. Clinical Implications: From Gene Polymorphisms to Patient Stratification

The clinical heterogeneity in SLE is likely attributable to inherent molecular heterogeneity that may affect therapeutic approaches. In recent years, this issue has begun to be tackled primarily through gene expression, autoantibody profiles, and cytokines to categorize people with SLE who exhibit unique molecular disease processes. Employing genetic information to stratify patients would benefit from the establishment of durable molecular markers for early classification [16]. Although significant progress has been made in recent years in linking genetic variations associated with disease to cellular functions, little of this understanding has been applied to the development of novel treatment approaches [53]. SLE varies in organ involvement, severity, and immunopathogenesis, which makes choosing the right therapy challenging. Anti-inflammatory and immunosuppressive medications are commonly used to manage immune issues in SLE. These include non-specific treatments, such as antimalarials, glucocorticoids (GCs), non-corticosteroid immunosuppressants, and targeted therapies. Patients with SLE often face a poor long-term prognosis and early death due to organ dysfunction. Over 50% develop organ damage within 10 years, with 30–50% experiencing it within the first 5 years. Damage accumulates over time, affecting multiple organ systems and leading to significant morbidity and mortality. The most commonly impacted systems are cardiovascular, neuropsychiatric, musculoskeletal, and renal, with gradual deterioration over 10–15 years [54].

Intracellular signaling pathways can be targeted therapeutically to treat autoimmunity, as demonstrated by the effectiveness of small-molecule JAK inhibitors (Jakinibs) in treating RA. Several Jakinibs are currently in preclinical development or undergoing clinical trials in patients with SLE [53]. The JAK-STAT pathway plays a key role in SLE development, with the *STAT4* rs7574865 polymorphism increasing IFN-γ production and leading to severe LN. Patients with the *STAT4* risk allele have shown improved outcomes with JAK inhibitors, such as tofacitinib, suggesting the potential for genetically tailored therapy [50]. The first drug to enter clinical trials, alvocidib, a first-generation pan-CDK inhibitor, can prevent interferon-γ-mediated nitric oxide (NO) production in vascular endothelial cells. It also inactivates *STAT1* in the JAK/STAT pathway and its downstream target, the interferon-γ response factor (*IRF1*), further confirming its anti-inflammatory effects and potential to reduce tissue damage in patients with SLE [55].

Strategies targeting T1FNs to mitigate autoimmune responses and lessen SLE severity have been developed for SLE treatment. Litifilimab, an antibody targeting blood DC antigen 2 (BDCA2), also known as CD303, which is exclusively expressed on pDCs and serves as a significant predictor of T1FN production, reduced the expression of genes related to IFN signaling in patients with SLE during a phase I clinical trial (NCT02106897), and decreased the activities of SLE and cutaneous lupus erythematosus in a phase II clinical trial (NCT02847598) [56].

The effectiveness and safety of anifrolumab, an IFN-I receptor monoclonal antibody, in patients with moderate-to-severe SLE across various clinical and genetic subgroups are outlined in a pooled post hoc analysis of the phase III TULIP-1 and TULIP-2 studies. The results confirm anifrolumab’s consistent efficacy and safety profile across diverse patient populations. The most notable difference in treatment benefit was observed in subgroups related to the drug’s mechanism of action, specifically patients with a heightened TIFN gene signature (IFNGS-high), which is the primary genetic and mechanistic target of anifrolumab, and patients with one or more abnormal baseline serological markers (e.g., anti-dsDNA positive, or low C3/C4). For example, the treatment difference in the British Isles Lupus Assessment Group-based Composite Lupus Assessment (BICLA) response at week 52 was greater for IFNGS-high patients (18.2%, nominal *p* < 0.001) than for IFNGS-low patients (9.3%, nominal *p* = 0.292). This indicates that identifying patients with an increased IFNGS, relevant to anifrolumab’s targeted mechanism, may help pinpoint those who derive greater benefit from the treatment, thereby linking the *IFNGS* gene signature to treatment response in SLE [57].

A few years ago, Apaer and colleagues thoroughly examined the pathogenesis of SLE through an extensive analysis of SLE-related data from the GEO database, with a particular focus on immune cell infiltration and the role of the ISG15 gene. The study found that *ISG15* plays a significant role in SLE, potentially exacerbating inflammatory responses and tissue damage via multiple pathways, including mitochondrial dysfunction, the DNA damage response, and the amplification of TIFN responses. Additionally, using molecular docking, the researchers demonstrated that *ISG15* can bind to genistein and alvocidib, two drugs known for their anti-inflammatory and immunosuppressive effects, respectively. This reveals new potential targets for SLE treatment. These findings not only enhance our understanding of the causes of SLE but also offer important insights for developing new therapeutic strategies [58].

Another potential therapeutic drug, iberdomide, an oral high-affinity cereblon ligand, promotes the degradation of the transcription factors Aiolos (*IKZF3*) and Ikaros (*IKZF1*), both of which are linked to the pathophysiology of SLE and immune cell development. In a study involving 42 patients with active SLE, iberdomide was generally well tolerated during the 12-week dose-escalation phase, followed by an open-label extension phase. Most treatment-emergent adverse events (TEAEs) were mild to moderate and commonly included upper respiratory tract infections, nausea, and diarrhea. Pharmacodynamic analysis showed dose-dependent reductions in blood levels of total B cells and pDCs. At the same time, exploratory efficacy endpoints indicated directional clinical activity, with improvements observed in the Physician’s Global Assessment (PGA) and Cutaneous Lupus Erythematosus Disease Area and Severity Index (CLASI) activity scores. Overall, the study’s findings support continued clinical research on iberdomide for SLE treatment by demonstrating a favorable benefit/risk balance [59].

**Figure 2:**
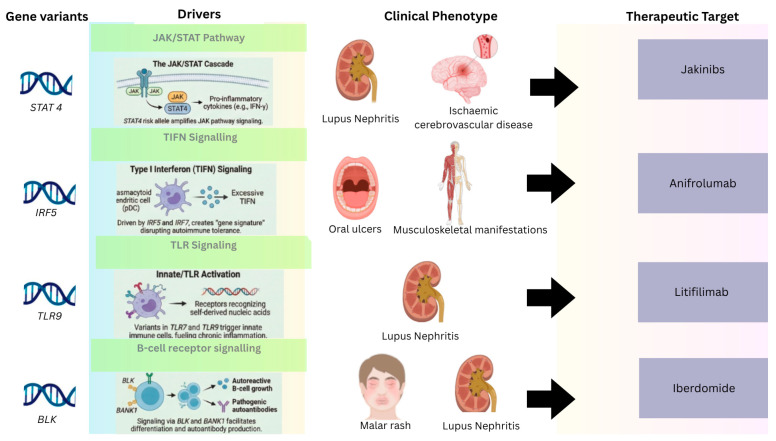
SLE immunopathogenesis: From genetic variants to targeted therapy. SLE drives immune dysregulation through key pathways, such as JAK/STAT, Type I (IFN), TLR signaling, and B-cell receptor signaling. Specific genetic variants, including *STAT4*, *IRF5*, *TLR7/9,* and *BLK,* are directly linked to clinical phenotypes, such as LN and neuropsychiatric symptoms, providing a foundation for targeted therapies, such as anifrolumab, Jakinibs, iberdomide, and litifilimab.

Although numerous clinical studies have been conducted to date, developing new pharmaceuticals targeting SLE remains difficult. The challenges in advancing SLE treatments include the heterogeneous pathophysiology of SLE, which involves multiple biological pathways in the disease process. Additionally, there are no specific biomarkers that can predict disease progression or response to therapy. Finally, the placebo response rate tends to be high due to concurrent oral glucocorticoid use in clinical studies. These factors make it harder to assess the potential clinical effectiveness of investigational medications accurately [25]. Figure 2 summarized complete translational trajectory of SLE pathogenesis linking genetic variants to molecular pathways, clinical phenotypes and therapeutic targets.

### 2.6. Utilizing Genetic Polymorphisms as Biomarkers

In recent years, biomarkers have become routinely used to diagnose, forecast, evaluate, and manage conditions such as SLE, diabetes, heart disease, cancer, and other illnesses. They can detect genetic variations, track gene or protein expression, monitor disease progression, and assess treatment responses. All of these can objectively predict difficult-to-observe biological traits. The 2019 EULAR/ACR SLE classification standard, which is based on SLICC-2012 and ACR-1997 and exhibits higher sensitivity and specificity, is currently the most widely used diagnostic criterion worldwide for SLE [8]. Some types of organ damage require an invasive method, such as a renal biopsy, for diagnosis, which causes significant pain to the patients. Consequently, diagnosing SLE is a time-consuming, complex, and laborious process, making the development of genetic markers for SLE diagnosis necessary. Given the varied nature of SLE and emerging evidence of the influence of geoepidemiology and epigenetics, it is not surprising that several biomarkers, such as *HLA-DRB1* and *STAT4,* have been identified. These anticipated variations make it challenging to translate association findings into reliable predictive biomarkers. Future technological advances in identifying novel approaches for SLE diagnosis will enable a more precise delineation of the relationships between genetic diversity and SLE susceptibility.

SNPs can serve as stable, objective germline markers that establish a fundamental framework for diagnosing and stratifying patients with SLE. Genetic polymorphisms provide reliable indicators for evaluating long-term risk and clinical progression, unlike transient molecular markers, such as serum cytokines or miRNAs, which fluctuate with disease activity and treatment. This focus on fixed germline variations responds to the urgent clinical need for persistent molecular markers to distinguish disease subsets, regardless of the patient’s current inflammatory state. The synthesis of high-resolution genetic data reveals significant correlations between specific susceptibility loci and distinct clinical manifestations, thereby providing clinicians with predictive tools for disease severity and organ-specific involvement. For example, the *HLA-DRB1* locus remains the most important genetic determinant of SLE. Particularly, the HLA-DRB1*03:01 (DR3) and HLA-DRB1*15:01 (DR2) alleles are highly indicative of severity. Proliferative nephritis (Class III and IV), anti-dsDNA, and anti-Ro/SSA autoantibodies are all strongly associated with DR3. Conversely, DR2 acts as a marker for the production of anti-Sm autoantibodies, oral ulcers, and earlier disease onset.

*STAT4* polymorphisms, especially the risk variant rs7574865, are dependable markers of severe phenotypes. These variants are linked to an increased risk of stroke, significant renal insufficiency, and early disease onset. They enhance IFN-γ production, connecting the stable genetic risk to harmful inflammatory signaling. *IRF5* expression levels are strongly associated with systemic inflammatory burden, showing a positive correlation with ESR and a negative one with hemoglobin levels. Elevated *IRF5* expression is a notable sign of active renal involvement. Similarly, *TLR7* rs3853839 serves as a biomarker for clinical symptoms, including thrombocytopenia and mouth ulcers.

Despite extensive data supporting the potential of new biomarkers, ongoing research is developing innovative tools for diagnosing, staging, and managing patients with SLE. More reliable immunological biomarkers, including both organ-specific and non-organ-specific SLE markers, are needed to better understand disease progression in SLE. Because no single biomarker has sufficient sensitivity and specificity for SLE, combining multiple biomarkers with mathematical models may be an effective way to evaluate the disease. Furthermore, advanced computer techniques are crucial for analyzing large datasets and identifying new biomarkers.

### 2.7. Limitations and Future Perspectives of SLE Genetic Stratification in Clinical Translation

Although GWASs have been transformative, identifying over 300 risk-associated SLE gene loci to date, the transition from discovery to clinical utility remains challenging due to the “GWAS Paradox.” The overwhelming majority of these SNPs confer only a marginal increase in individual disease risk, despite the sheer number of associations. These polymorphisms have minimal clinical value for disease identification or diagnostic screening when considered in isolation, as they account for only a fraction of the intricate pathogenic landscape.

The high degree of replication variability across distinct ancestral cohorts is a critical obstacle to the clinical application of these findings. While certain main loci, such as those within the MHC, exhibit robust associations globally, numerous other variants show significant population-specific frequencies. Alleles that confer significant risk in one population, for example, *HLA-DR3* in Europeans, may exhibit distinct associations in Asian cohorts, where *HLA-DR2* is frequently the predominant allele. Variations in risk allele frequencies and geoepidemiological factors account for these discrepancies. This variability, which is often shaped by distinct linkage disequilibrium patterns and gene–environment interactions, impedes the direct transmission of genetic risk profiles between populations. Consequently, the field is transitioning from the “one SNP one phenotype” paradigm to more comprehensive genomic assessments.

The polygenic risk score (PRS) is a numerical score that measures an individual’s genetic susceptibility to specific traits or diseases by analyzing multiple genetic variants across the genome. PRSs can predict the risk of developing specific conditions, making them essential tools for personalized treatment and risk assessment. Limited research has examined the PRS for SLE and its association with disease manifestations or severity; however, these studies indicate that individuals with a high PRS for SLE are more likely to exhibit severe phenotypes, early onset, and increased mortality. In contrast to individual SNPs, PRSs consider the aggregate genetic load across numerous loci. Integrating these ratings with multi-omics data (proteomics and transcriptomics) via AI could enhance patient classification and treatment response prediction [60].

Longitudinal validation studies are necessary to assess the predictive power of genetic markers. Moreover, several ethical considerations must be addressed to ensure the responsible implementation of precision medicine as genetic stratification transitions from research to clinical practice. Initially, it is critical to ensure that genomic biomarkers and targeted therapies are accessible to a wide range of socioeconomic and ethnic groups to prevent the exacerbation of preexisting health disparities. Because SLE exhibits substantial geoepidemiological variability, it is ethically necessary to develop genetic risk models using multiethnic cohorts. Otherwise, precision medicine may inadvertently exclude underrepresented populations. Additionally, protecting sensitive biological information requires robust frameworks for data privacy and informed consent to integrate multi-omics and large-scale genetic datasets.

## 3. Conclusions

Genomic research has uncovered critical disease pathways involving failures in self-antigen clearance, innate immune responses to autoantigen-related damage, and the generation and persistence of autoreactive lymphocytes. Future research is expected to deepen our understanding of the mechanisms behind disease development and manifestation, clarify disease heterogeneity, explore its connections with other autoimmune disorders, and assess how genetic diversity influences disease prevalence and severity. Identifying and mapping all SNPs associated with drug responses in SLE is vital for the success of autoimmune treatments, as it can reduce or prevent drug-related issues, such as adverse drug reactions (ADRs), and enhance therapeutic effectiveness. Studying genetic variants in SLE has become a fundamental part of personalized medicine, enabling customized therapies based on genetic profiles. Consequently, strengthening the link between genetic diversity and biological mechanisms is likely to lead to more effective strategies for disease prevention and the minimization of therapy-related damage.

## Figures and Tables

**Table 1:** Summary of gene variants involved in innate and adaptive immune pathways associated with SLE clinical phenotypes.

Innate and Adaptive Immune Pathway	Gene Variants Involved	SLE Phenotypic Features/Clinical Manifestation	Author
TLR signaling	*IRF5*, *IRF7*, *TLR9*	LN	[10,11,29,30]
T1FN signaling	*ACP5*, *IRF5*, *IRF7*, *IRF8*, *STAT4*, *PTPN22*, *OPN*/*SPP1*, *IFIH1*, *TYK2*, *TLR7*, *TLR8*, *TLR9*	Spondyloenchondrodysplasia (SPENCD), dermatologic, musculoskeletal, renal, vascular, central nervous system and hematological manifestations, premature atherosclerosis, and Neuropsychiatric SLE (NPSLE)	[25,31,32,33]
Intracellular signaling pathway—JAK-STAT pathway	*STAT4*, *PTPN2*	LN/renal involvement, oral ulcers, ischemic cerebrovascular disease, and serositis	[10,11,30]
B-cell receptor signaling	*BANK1*, *RASGRP3*, *BLK*	LN, malar rash, discoid lesions, and serositis	[3,11,30]
NF-κB signaling	*TNIP1*	LN	[11]
Th17 pathway	*ITGAM*, *FCGR2A*, *FCGR3A*, *STK17A*	Joint involvement, and skin involvement	[10]
IL-17 pathway	*TRAF3IP2*	Pericarditis	[10]
cGAS-STING pathway	*TREX1*	Neuropsychiatric SLE (NPSLE)	[10]

**Table 2:** A summary of the associations between genetic variants and organ and system involvement in SLE across different ancestries.

Gene	SNP	Disease Phenotype	Population	OR	*p* -Value	Author
HLA Class II	rs9270970 (AG)	Fibrosis-associated inflammation	Thai	1.82	3.61 × 10^−26^	[45]
*IRF5*	rs4728142	Hematological condition	Malaysian Chinese	5.61	0.005	[34]
*SLC12A1*	rs1878186	Mucocutaneous manifestation	Malay	2.01	0.008	[34]
*RNU6-546P*	rs4544377	Mucocutaneous manifestation	Malaysian Chinese	2.82	0.023	[34]
*STAT 4*	rs7582694 (CG)	LN	Thai	1.57	8.21 × 10^−16^	[45]
*TNFSF4*	rs2205960	LN	European	1.14	0.0013	[11]
*TLR7*	rs3853839 (CG)	Oral ulcer	Egyptian	0.25	0.01	[44]
*GTF2I*	rs73366469 (AG)	LN	Thai	1.73	2.42 × 10^−11^	[45]
*IL-10*	IL-10 -1082 (AA)	Immunologic disorder	Indian	1.6	0.1880	[46]
*ITGAM*	rs1143679 (AG)	Oral ulcers, LN	North Indian	1.73	0.001	[47]
*FBN2*	rs74989671 (AG)	Connective tissue disorders	Thai	1.54	1.61 × 10^−8^	[45]
*TNFAIP3*	rs5029939 (GC)	N/A	North Indian	1.91	0.01	[47]
*SIGIRR*	rs7396562	Mucocutaneous manifestation	Han Chinese	1.36	0.05	[48]
*TNIP1*	rs7708392 (CG)	Renal disorder	Egyptian	5.05	0.001	[49]
*BLK*	rs7812879	Renal disorder	Chinese	1.55	0.02	[50]
*NCF2*	rs17849502	Juvenile autoimmune rheumatic diseases	Belarusian	2.60	0.023	[51]
*IKZF1*	rs4917014	Hematological disorder	Chinese	0.75	0.005	[42]
*TNFSF4*	rs1234315	Arthritis	Han Chinese	1.45	1.5 × 10^−16^	[30]
*RUNX3*	rs4649038	Musculoskeletal manifestations	Han Chinese	1.13	0.009	[6]
*ISG15*	N/A	Lymphocytopenia	Kazakhs	N/A	N/A	[52]
*SLC5A11*	N/A	Serositis	Kazakhs	N/A	N/A	[52]

## Data Availability

No new data were created or analyzed in this study. Data sharing does not apply to this article.
